# Outcome of Surgical Treated Isolated Pronator Teres Syndromes—A Retrospective Cohort Study and Complete Review of the Literature

**DOI:** 10.3390/ijerph19010080

**Published:** 2021-12-22

**Authors:** Harald Binder, Armin Zadra, Domenik Popp, Micha Komjati, Thomas M. Tiefenboeck

**Affiliations:** 1 Department of Orthopaedics and Trauma Surgery, Division of Trauma Surgery, Medical University of Vienna, 1090 Vienna, Austria; harald.binder@meduniwien.ac.at (H.B.); domenik.popp@meduniwien.ac.at (D.P.); 2 LKH Südsteiermark, Department of Orthopaedics, Bad Radkersburg, 8490 Südsteiermark, Austria; zadra-handchirurgie@aon.at; 3 First Department of Orthopaedics, Hospital of sacred Heart of Jesus, 1030 Vienna, Austria; micha.komjati@kh-herzjesu.at

**Keywords:** pronator teres syndrome, clinical outcome, decompression, review of the literature

## Abstract

Purpose: This study aims to elucidate the occurrence of postoperative carpal tunnel syndrome (CTS), the functional outcome of patients with primary pronator teres syndrome (PTS), and review complete literature regarding this topic. Material and Methods: A retrospective chart review was conducted in patients with PTS at a single center. In all patients, a numeric Visual Analog Scale (VAS) score, Pinch-Test, Jamar hand dynamometer test (JAMAR), and the Disabilities of the Arm Shoulder and Hand (DASH) score were analyzed preoperatively and at final follow-up to assess outcome. Additionally, a complete review of the literature was performed, including all data dealing with pronator teres syndrome. Results: Ten female and two male patients were included with a mean age of 49 years. Significant improvement in DASH and numeric VAS was detected at latest postoperative follow-up. In three patients, clinical signs of CTS pathology were detected during the follow-up period. One patient needed to be treated surgically, and in the other two patients, a conservative management was possible. In one patient (8%), a PTS recurrence was detected. All patients presented satisfied at latest follow-up. Conclusion: In one-fourth of our patients, a CTS occurred during the follow-up period. Therefore, focusing on double-crush syndrome in unclear or mixed symptoms is necessary to avoid multiple operations. Furthermore, it seems that assessment with NCV is not enough for diagnosing PTS; therefore, further research is needed to clarify this problem.

## 1. Introduction

The pronator teres syndrome (PTS), first described by Seyfarth in 1951 as a representative proximal forearm median neuropathy [1], is a very rare and rather difficult to assess pathologically. The most common compression of the median nerve (MN) occurs when it passes between the humeral and ulnar heads of the pronator teres muscle (PTM), through the so called pronator canal, and is manifested by symptoms such as painfulness of the PTM and paraesthesia, dysesthesia, or even paralysis in the MN innervation zone [2,3,4,5].

The low incidence of PTS, with a rate of 1 to 5% [6,7,8,9,10] of all median neuropathies, makes this a very seldom disease with limited data available. Furthermore, not only is the diagnosis tricky in differentiating it from other median neuropathies, but providing a proper treatment strategy can also be challenging.

Usually, the cause of compression in the pronator canal is muscle hypertrophy due to excessive overloading or trauma with resulting hematoma or even deformity [2,3,4]. It is also associated with the presence of variable anatomical structures at the starting point of the humeral head of the PTM, i.e., a supracondylar process or an anomalous Struthers ligament [11].

Diagnosing PTS is difficult, and therefore, a various number of pronator aggravation tests are described. However, a negative carpal steroid injection can help support the diagnosis [12]. Electrodiagnostic studies for PTS present positive in only 7–31% [13] of the patients, which stands in contrast to CTS, where 84% are positive. Therefore, electrodiagnostic studies might play an important role to rule out other compression syndromes; however, they play a limited role for diagnosing PTS [12].

Additionally, ultrasound and MRI imaging can be used in specific cases, such as hematoma causing compression or oedema of the muscle as early denervation signs, for example [14].

Decision and timing of surgery in treating PTS can be challenging and is based mainly on the clinical presentation. Clinical presentations showing severe thenar muscle atrophy (particularly with regard to a short period of time, indicating progression of axonal loss) as well as a failed physical therapy and a progression of symptoms with a significant deterioration in the quality of life are the main criteria of the surgical approach [15].

The purpose of this study was to review experience with PTS at a single center. Furthermore, this study aims to elucidate the occurrence of postoperative CTS and the functional outcome of this patient collective.

## 2. Materials and Methods

### 2.1. Retrospective Cohort

A retrospective chart review was conducted in patients with PTS treated between 2005 and 2017 in a single-center institution. Patients were identified by a modified electronic search of the computerized patients’ record system, of (blinded for review). The medical case files and relevant radiological studies (standardized x-rays in a/p and lateral view just as ultrasound and/or MRI in case of suspected vascular malformation or tumour growth) as well as all neurological examinations, including nerve conduction velocity (NCV) tests of all identified patients, were retrospectively analyzed. Additionally, age, follow-up, numeric VAS score, PINCH, JAMAR, and DASH score were analyzed preoperative and at final follow-up.

Inclusion criteria were as follows: (1) positive preoperative evaluation in patients with at least one of these three methods: (1.1.) positive Tinel’s sign at pronator area, (1.2.) positive resisted forearm pronation test (compressed by pronator muscle), (1.3.) positive middle-finger FDS (flexor digitorum superficialis) test, and (1.4.) positive resisted flexion supination test (compressed by lacertus fibrosus) [13,14]; (2) the need for surgical intervention; (3) over 18 years of age; and (4) failed conservative management.

Exclusion criteria included the following: (1) patients with negative preoperative evaluation with regards to PTS provocation tests; (2) additional injuries to the cervical spine including radiculopathies and myelopathies as well as spinal stenosis and/or spinal disc herniation; and (3) patients under 18 years of age.

Surgical procedure: In all patients, a standard 6.0- to 8.0-cm bayonet-shaped incision 3 cm above the elbow crease over the flexor and pronator muscles ulnar to the biceps muscle was performed. The lacertus fibrosus and brachial sheath were identified and both cut. Thereafter, the median nerve was identified followed by a release of any fascial bands and above the nerve. Full decompression was achieved by releasing thickened and tight superficial fascia of the pronator teres muscle and stretching of the tendon origin. There was no need for an elongation or a second incision because preoperative x-rays showed no supracondylar spurs in any of the patients.

Complications, such as infection (swelling, redness, wound breakdown, discharge, or positive culture with a pathogen), bleeding, and neurologic deterioration were defined as major complications.

Postoperative functional and neurological outcomes were assessed during the routine follow-up examination by the senior orthopaedic surgeon who also performed all the surgical procedures. Pre- and postoperative evaluation of functional outcome included the DASH score and range of motion as well as strength assessment using PINCH and JAMAR. Neurological evaluation included clinical evaluation of neurological symptoms such as skin sensitivity or Hofmann–Tinel sign, for example. Prior to the investigation, the corresponding institutional review board (Medical University of Graz) approved the study (EK NR29-608ex16/17). Prior to study inclusion informed consent was obtained from all included patients at time of follow-up.

### 2.2. Statistics

First, data were tested for normal distribution by using the Shapiro–Wilk’s test, then homogeneity was tested with the Levene test. When both assumptions were applied, a mixed-model ANOVA was used to test differences between the two groups. For all tests, *p* < 0.05 was considered significant. Descriptive statistics, including means, median, range and standard deviations, were performed for the two groups. All calculations were made using Microsoft Excel^®^, SPSS^®^ software (Version 25.0, SPSS Inc., Chicago, IL, USA).

### 2.3. Review

The initial research was conducted by three reviewers, who were blinded for the review, on the 26th and 29th of November 2021 using the most common electronic data bases (Medline, PubMed, Cochrane Library and Embase). A total of 180 studies were identified and selected for further manual evaluation. To make the retrieval of studies as reproducible as possible, the following keywords were used: pronator teres syndrome, median neuropathy, anterior interosseous syndrome, and medianus entrapment.

The references of the included full-text articles were cross-checked for additional studies that met the inclusion criteria as well.

#### 2.3.1. Inclusion Criteria

Level of evidence I to IV studies were included. The literature search was focusing on clinical occurrence of pronator teres syndrome (PTS) in comparison to carpal tunnel syndrome (CTS), possible anatomical causes of this entity, and approaches to conservative and surgical treatment. Diagnostic, prospective, retrospective, cohort, and cross-sectional studies comparing different diagnostic techniques and published in peer-reviewed scientific journals were included in this review. Due to a lack of literature, case reports were included as well as only publications written in English and German with available full-text.

#### 2.3.2. Exclusion Criteria

Non-clinical studies regardless of type were excluded. Furthermore, non-English, non-German, not available in full-text, lower-quality randomized trials not matching with established standards of level I–IV, articles not evaluating the PTS, or not meeting a validated gold standard were excluded from this review.

#### 2.3.3. Study Selection

The second step was established on pre-selection, implementing the exclusion criteria in general, and identifying randomized trials, like obvious irrelevance for the topic (mammal cadaver studies, only about CTS, poor structure, and other cubital entrapment syndromes).

The third step of this process consisted of manually screening titles and abstracts to focus on relevant studies about the PTS and exclude studies with incompatible study design.

Finally, the remaining full-text articles were independently evaluated from the two above-mentioned reviewers based on the inclusion criteria. In case of disagreement between the authors, the lead author was responsible for further decision making.

#### 2.3.4. Bias

Many of these studies show several sources of possible bias. Not a single article had specific bias tools implemented, and the majority of literature consists of single case reports. This can distort the interpretation of our findings. In all evaluated studies that contained the highest levels of methodical structure, blinding procedures were not stated. Partial verification bias, incorporation bias, and selection bias occurred in almost all included studies.

## 3. Results

**Figure 1:**
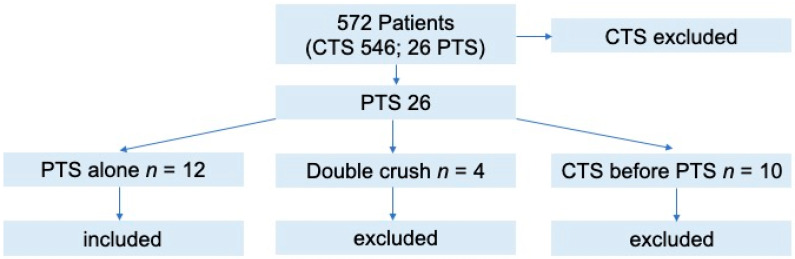
Patient flow-chart.

Twenty-six patients out of 572 with peripheral nerve compression syndromes (CTS, PTS, double crush) were identified with PTS symptoms at the orthopaedic ward, presenting an overall incidence of PTS of 4.5%. This group consisted of four primary double-crush syndromes, ten patients who previously presented with treated CTS and underwent additional treatment for PTS, and twelve primary PTS cases. Therefore, after exclusion, twelve patients were finally included (Figure 1. Presents patient flow-chart).

The mean age at time of surgery was 49 years (median; 42 years, range; 32 to 74 years, STD 11.4 years). Ten female and two male patients were included. Nerve conduction velocity (NCV) studies were performed in all patients (100%) and showed positive signs for CTS/PTS in nine patients (75%), whereby three patients (25%) had a negative outcome. All patients showed a positive resisted forearm pronation test, 10 patients presented a positive middle finger FDS (flexor digitorum superficialis) test (compressed by FDS arch), and seven patients showed a resisted flexion supination test (compressed by lacertus fibrosus). Only three patients were positively tested for Tinel’s sign, whereas no patient showed a positive Phalen test (Table 1 presents the baseline demographics; Table 2 presents the detailed overview of clinical presentation).

During follow-up, in three patients, all female, clinical signs of CTS pathology were detected. Symptoms occurred after a mean duration of 17 months (median; 20 months, range; 9 to 22 months, STD 6 months). In all of these patients, a positive NCV for CTS was demonstrated at time of treatment. One patient needed to be treated surgically; in the other two patients, a conservative management was possible. These patients presented free of CTS symptoms at latest follow-up.

In one patient (8%), a PTS recurrence was detected 12 months after primary surgery, and following revision surgery, no PTS signs were present anymore. This patient also suffered from a localized subcutaneous infection with Staphylococcus epidermidis. A rapid intravenous antibiotic therapy with clindamycin resulted in the patient eliminating the superficial infection.

The mean follow-up time in all patients was 75 months (median; 67 months, range; 32 to 120 months, STD; 30 months). Preoperatively assessed scores compared to postoperative scores are shown in Table 3.

Significant changes in DASH and numeric VAS were found between preoperative and last follow-up assessment. PINCH and JAMAR scores revealed no significant changes; however, an improvement in scores could be demonstrated.

Mean duration till surgical procedure was in mean six months (median 6 months; range; 3 to 12 months; STD 3 months).

All patients (100%) affirmed the positive effect of the operation and would do it again in case of symptoms.

**Figure 2:**
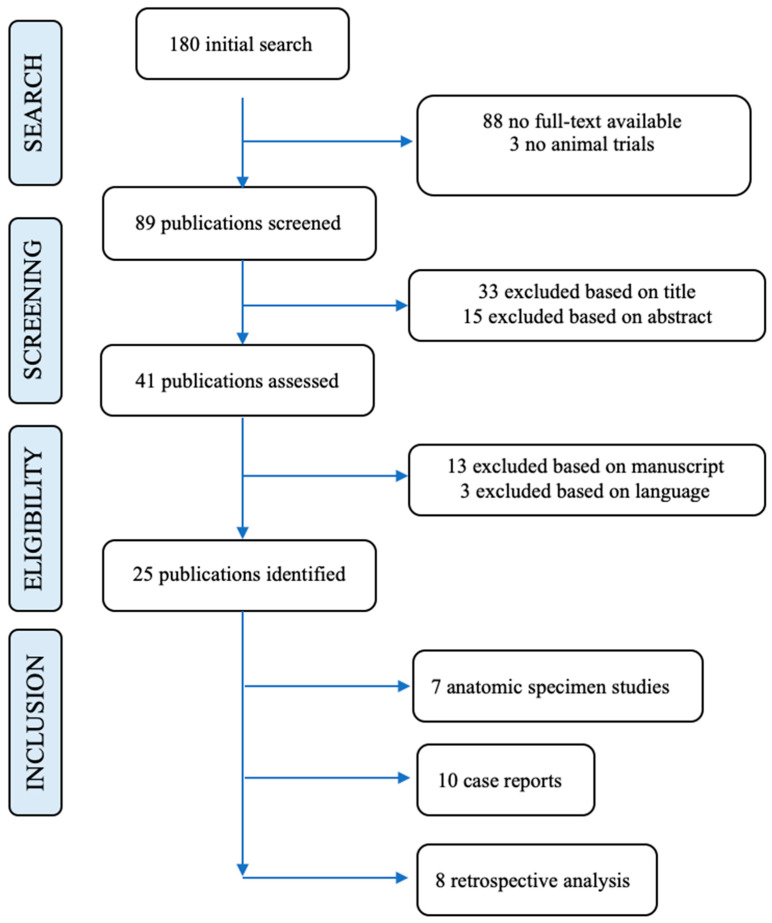
Represents the literature review of pronator teres in detail.

Results of the literature review:

Finally, 25 studies could be included in this review. Seven anatomical specimen studies, 10 case reports, and eight retrospective analysis were included (see Figure 2. Literature review in detail). We concluded that there is no evidence in treatment and also no evidence regarding a diagnostic algorithm.

## 4. Discussion

Isolated PTS is reported very rarely in literature. Often, misdiagnosis caused by overlapping symptoms of CTS and PTS leads to insufficient surgical and clinical outcome. In addition, literature states the occurrence of double-crush syndrome in varying numbers of up to 73%, making its diagnosis very difficult. The diagnosis of PTS/CTS and double-crush syndrome is mainly a clinical one [12] and can be supported by NVC [13], the way it was done in our patients. Our standardized tests in all patients included the resisted forearm pronation test (compressed by pronator muscle), middle-finger FDS (flexor digitorum superficialis) test (compressed by FDS arch), and the resisted flexion supination test (compressed by lacertus fibrosus), which is also presented in literature as the standard clinical diagnostic method [14,15]. In all of our patients, the resisted forearm pronation test (compressed by pronator muscle) was positive. However, Tinel’s sign was only positive in three patients. In the initial examination, Phalen’s manoeuvre in all patients was negative. Nevertheless, one-fourth of our patients presented with a postoperative CTS within two years after PTS surgery. It needs to be mentioned that all of these patients initially presented without any clinical signs of CTS, demonstrating the difficulty of primarily diagnosing a double-crush syndrome. Furthermore, it needs to be mentioned that this cohort is almost in their fifties and mainly female and therefore prone to getting CTS. Due to this fact, differentiation between a primary missed double-crush syndrome and a newly developed CTS is difficult. However, caused by these overlapping symptoms, it needs to be discussed if the PTS symptoms play a dominating role leading to the possibility of overlooking CTS and therefore the pathology of a double-crush syndrome or whether it is just coincidence. There is no evidence in literature regarding this phenomenon, and this study also lacks evidence, caused by the low number of included patients. Incidence of double-crush syndrome is presented in literature, with an estimated range from 6.7% up to 73% [16,17]. However, isolated PTS presents very rarely with an estimated incidence of 0.4 to 6% [7,8,18]. This corresponds to our incidence of 4.5% in a 11-year observational period.

There are studies comparing the role of PTS in revision of carpal tunnel surgery [12] and studies investigating concurrent CTS and PTS [18]; however, there are fewer data focusing on long-term outcome and postoperative complications after treatment of isolated PTS.

In our collective, significant changes in DASH and numeric VAS were observed between preoperative and last follow-up assessment, which is comparable with presented results in literature [10,12,19]. We did not detect significant changes in the PINCH and JAMAR tests, which might be caused by the low number of patients. In summary, good to excellent mid- to long-term outcomes can be reached by surgical treatment of PTS with a low rate of postoperative complications. Due to the low number of cases, further multicentre studies will be needed to evaluate the risk of CTS occurrence after isolated PTS surgery. This is also supported by our review of the literature, where only 24 studies could be finally included (Table 4. presents an overview of clinical studies and Table 5. presents an overview of diagnostics).

## 5. Conclusions

In conclusion, one-fourth of our patients presented with CTS during follow-up. Therefore, focusing on double-crush syndrome and mixed symptoms is necessary to avoid multiple operations in this patient collective. Furthermore, it seems that assessment with NCV is not enough for diagnosing PTS; therefore, further research is needed to clarify this problem.

### Limitations

Firstly, readers have to be aware of the retrospective character of this study and the limitations associated with this study design. Secondly, there was a lack of a control group due to the rareness of this disease. Another limitation is the small number of patients and therefore a lack of evidence. However, this is one of the first studies presenting long-term outcomes after primary PTS.

## Figures and Tables

**Table 1:** Baseline demographics of patients with PTS alone.

Variables	
*n*	12
median age (yrs.)	42
sex (m/f)	2/10
side (r/l)	9/3
**Clinical presentation**	
tenderness	12
resisted forearm pronation test	12
positive FDS test	10
flexion supination test	7
Hofmann–Tinel positive	3
NLG	9
CTS yes/no	0

*n*, number of patients; m, male; f, female; r, right; l, left.

**Table 2:** Clinical presentation of patients in detail.

Pat. No.	Time Period Preoperative Symptoms	Muscle Atrophy Preoperative	Muscle Strength Preoperative	Age	Sex	Dominant Hand	Disabled
1	5 m	no	4+	38	female	yes	no
2	12 m	yes	4−	45	male	no	no
3	6 m	no	5	50	female	yes	no
4	3 m	no	5	60	female	yes	no
5	12 m	yes	3	32	female	yes	yes
6	12 m	yes	4	74	female	yes	yes
7	2 m	no	5	65	female	no	no
8	3.5 m	no	5	40	female	yes	yes
9	4 m	no	4+	40	female	no	yes
10	6 m	no	4−	39	female	yes	yes
11	12 m	no	4	55	male	yes	yes
12	12.5 m	yes	3	50	female	yes	yes

**Table 3:** Clinical outcome of patients pre- vs. latest follow-up.

**Pat. No.**	**Pre Operative**	**Latest FUP**	**Pre Operative**	**Latest FUP**	**Pre Operative**	**Latest FUP**	**Pre Operative**	**Latest FUP**
	DASH		PINCH		JAMAR		numeric VAS (0–10)	
1	60.8	0.00	2.5	9.5	4.3	26.0	7	0
2	54.1	24.2	5	7.0	16	27.3	7	0
3	27.5	20.8	4	8.0	13	42.0	8	0
4	43.3	15.0	7	5.0	26	26.0	6	0
5	55.0	58.3	4.5	5.0	21	22.0	10	5
6	79.2	58.3	2.5	2.0	9.5	14.0	9	0
7	55.0	16.7	5	12.0	26	32.0	6	1
8	29.2	5.8	5.5	11.0	29	18.5	8	0
9	73.3	12.5	10	13.0	28	30.0	8	0
10	57.5	39.2	6.5	2.5	25	6.0	9	2
11	38.3	9.2	6.5	5.5	38	26.0	8	0
12	55.8	14.2	4.2	6.0	15	28.0	7	0
Mean (Median, Range, STD)	48.7 (52.4; 13.3 to 79.2; 15.5)	22.8 (15.8; 0 to 58.3; 18.4)	5.3 (5.0; 2.5 to 10.0; 2.0)	7.2 (6.5; 2 to 13; 3.4)	20.9 (23; 4.3 to 38; 9.15)	24.8 (26.0; 6.0 to 42.0; 8.7)	8 (8; 6 to 10; 1)	0.7 (0; 0 to 5; 1.4)
*p*-Value	*p* < 0.01. *p* = 0.083. *p* = 0.347. *p* < 0.01							
**Pat. No.**	**Muscle Atrophy Latest FUP**		**Muscle Strength Latest FUP**		**Dysesthesia Latest FUP**	**Disabled**	**Postoperative Scar Tissue Impairment**	**Limited ROM**
1	no		4+		no	no	no	no
2	no		5		yes	no	no	no
3	no		5		no	no	no	no
4	no		5		no	no	no	no
5	yes		4−		yes	yes	yes	yes
6	no		4		no	no	no	no
7	no		5		no	no	no	no
8	no		5		no	no	no	no
9	no		5		no	no	no	no
10	no		4−		yes	no	yes	no
11	no		5		no	no	no	no
12	no		4+		no	no	no	no

ROM, range of motion; FUP, follow-up; Pat., patient; No., number.

**Table 4:** Overview of clinical studies—literature review.

Author	Cases/Side	Age	Occupation/Origin	Symptoms					Physical Examination
				PPF	GS	T	NB	HY	Findings
Lacey [19]	1; bilateral ♂	49	Office job Hobby carpenter	+	+	+	+		Forced supination FPL + FDP weak
Ashworth [20]	1; left, D ♂	36	Spontaneous	+	+	−	+		Normal reflexes
Tulwa [21]	1 ♀	54		+	−	−	−	Thumb Index Middle	US, X-ray, MRI normal
Lee [22]	1, right, D ♀	21	Spontaneous Office job	−	+	+	+	Thumb Index Middle	Thenar muscle atrophy (Flex: index + middle 3/5; thumb Abduction 3/5, Weak OK sign Normal reflexes
Danielsson [23]	1, left, D ♀	14	Previous osteotomy Congenital radio-ulnar synostosis	+			+	Thumb Index	Progression to total sensory and motor palsy
Morris [24]	7, D ♂	28–70 a Mean 47	Manual labour	3/7	3/7	7/7	6/7	6/7	Atrophy 1/7 Weakness FPL 7/7, OP 5/7 APB 6/7 FDP 3/7
Megele [25]	1, left ♂	59 a	After car tour	+	+	+	n.d.	n.d.	FPL strength 4/5
Bridgeman [5]	83			39%	49%	12%	58%	25%	2% nocturnal paresthesia; 11/16 initial CTS surgery without improvement
Zancolli [15]	1, bilateral mentioned 44 cases	71 a	Onset 2 a ago	+	n.d.	+	+	Thumb Index Middle	Nocturnal paresthesia
Hsiao [18]	21, 3♂/18♀	42–69 a mean 52 a		+			+		
Mujadzic [26]	61, 22♂,39♀	23–70 a	33 (54%) laborers 38 (62%) dominant hand	22 (36%)	12	n.d.	39/62 (64%)	Thenar	
Luangjarmekorn [12]	20, 14♀/3♂/3bilateral		Mean 53	20/20		19/20	15/20		
Sos [9]	55, 29♂/26♀/2bilateral	56 +/–15		32/55	44/55	11/55	39/55	29/55	Tinel pos: 15/55 elbow; 17/55 forearm; 5/55 wrist Amyotrophy 44/55

D, dominant hand; a, years; PPF, pain prox. forearm; GS, grip strength; T, tenderness; NB, numbness; Hy, hypoesthesia.

**Table 5:** Overview of Diagnostics—literature review.

Author	Tests	Treatment	Diagnostics	NCV	FU	Remarks
			EMG			
Lacey [19]	Tinel sign + Phalen + PT compression +	Initially conservative, after 4 months surgery	EMG: active denervation bilaterally in FPL	normal	4 years no symptoms	
Ashworth [20]	Tinel −	Surgery	Fibrillations	normal	6 w pop normal + EMG normal	
Tulwa [21]	Tinel − Phalen −	Surgery	Normal	delay	No symptoms	
Lee [22]	Tinel + PT + Resisted pronation+	Conservative: No improvement for 3 months surgery	Abnormal		1 d pop tingling sensation improved 1 m atrophy improved; Tinel −; 4 m Strength 4 +; No FUP EMG	MR abnormal high signal intensity on t2-weighed images
Danielsson [23]		surgery	N.d.			Osteotomy between origin and attachment of PT
Morris [24]		Conservative 5/7 (corticosteroid injection)	5/7 pos			
Megele [25]		Surgery 1 a after onset	Normal	normal	2 w full recover pop	
Bridgeman [5]	Tinel pos 7%	16 surgery	58/83 70% pos	25/83 (30%) motoric + 45/83 b (65%) sensoric +	8/16 improvement	9/16 fibrous band caused compression
Zancolli [15]	Tinel − PT compression test − Phalen −	Surgery	+	N.d.	Case: Left: 6 d pop no pain or numbness; Right: 18 d pop recover	44 Cases: 41 (93%) pop no more symptoms; 3 Pat still numbness
Hsiao [18]		Surgery	3/21 +	21/21	6 w, 3 m,12 m; 15/21 (71%) complete relief; 6/21 (29%) occasional paresthesia, no sensory deficits	
Mujadzic [26]		Mixed	12/61 +		Mean 7 m; 3–19 m; 39/61 (64%) complete relief, 13/61; 9 partial relief	
Luangjarmekorn [12]	Tinel 15/20 Phalen 19/20 PTS provocative test 19/20 Steroid injection Test	Surgery	1/20 +			Steroid injection CT 8/14 not improved
Sos [9]	Tinel 37/55 Weber Test 29/55	Surgery	52/55 +	43/55	84+/−70 m; 10 lost to FUP; 28/45 immediate recovery	18/45 diminished strength; 19/55 sensory signs

w, weeks; m, month; FU, follow-up; pop, postoperative; +, positive; −, negative.

## Data Availability

The datasets generated and/or analysed during the current study are not publicly available due to data privacy but are available from the corresponding author upon reasonable request.

## References

[1] Seyffarth H. (1951). Primary myoses in the M. pronator teres as cause of lesion of the N. medianus (the pronator syndrome). Acta Psychiatr. Et Neurol. Scand. Suppl..

[2] Andreisek G., Crook D.W., Burg D., Marincek B., Weishaupt D. (2006). Peripheral Neuropathies of the Median, Radial, and Ulnar Nerves: MR Imaging Features. Radiographics.

[3] Dang A.C., Rodner C.M. (2009). Unusual Compression Neuropathies of the Forearm, Part II: Median Nerve. J. Hand Surg..

[4] Miller T.T., Reinus W.R. (2010). Nerve Entrapment Syndromes of the Elbow, Forearm, and Wrist. Am. J. Roentgenol..

[5] Bridgeman C., Naidu S., Kothari M.J. (2007). Clinical and electrophysiological presentation of pronator syndrome. Electromyogr. Clin. Neurophysiol..

[6] Gessini L., Jandolo B., Pietrangeli A. (1983). Entrapment neuropathies of the median nerve at and above the elbow. Surg. Neurol..

[7] Hartz C.R., Linscheid R.L., Gramse R.R., Daube J.R. (1981). The pronator teres syndrome: Compressive neuropathy of the median nerve. J. Bone Jt. Surg. Am..

[8] Nigst H., Dick W. (1979). Syndromes of compression of the median nerve in the proximal forearm (pronator teres syndrome; anterior interosseous nerve syndrome). Arch. Orthop. Trauma Surg..

[9] Sos C., Roulet S., Lafon L., Corcia P., Laulan J., Bacle G. (2021). Median nerve entrapment syndrome in the elbow and proximal forearm. Anatomic causes and results for a 55-case surgical series at a mean 7 years’ follow-up. Orthop. Traumatol. Surg. Res..

[10] Asheghan M., Hollisaz M.T., Aghdam A.S., Khatibiaghda A. (2016). The Prevalence of Pronator Teres among Patients with Carpal Tunnel Syndrome: Cross-sectional Study. Int. J. Biomed. Sci. IJBS.

[11] Vymazalová K., Vargová L., Joukal M. (2015). Variability of the pronator teres muscle and its clinical significance. Rom. J. Morphol. Embryol..

[12] Luangjarmekorn P., Tsai T.M., Honsawek S., Kitidumrongsook P. (2016). Role of pronator release in revision carpal tunnel surgery. SICOT-J.

[13] Rodner C.M., Tinsley B., O’Malley M.P. (2013). Pronator Syndrome and Anterior Interosseous Nerve Syndrome. J. Am. Acad. Orthop. Surg..

[14] Spinner M., Spencer P.S. (1974). Nerve Compression Lesions of the Upper Extremity. Clin. Orthop. Relat. Res..

[15] Zancolli E.R., Perrotto C.J. (2012). New Mini-invasive Decompression for Pronator Teres Syndrome. J. Hand Surg..

[16] Upton A., Mccomas A. (1973). The double crush in nerve-entrapment syndromes. Lancet.

[17] Kane P.M., Daniels A.H., Akelman E. (2015). Double Crush Syndrome. J. Am. Acad. Orthop. Surg..

[18] Hsiao C.-W., Shih J.-T., Hung S.-T. (2017). Concurrent carpal tunnel syndrome and pronator syndrome: A retrospective study of 21 cases. Orthop. Traumatol. Surg. Res..

[19] Lacey S.H., Soldatis J.J. (1993). Bilateral pronator syndrome associated with anomalous heads of the pronator teres muscle: A case report. J. Hand Surg..

[20] Ashworth N.L., Marshall S.C., Classen D.A. (1997). Anterior interosseous nerve syndrome presenting with pronator teres weakness: A case report. Muscle Nerve.

[21] Tulwa N., Limb D., Brown R.F. (1994). Median Nerve Compression within the Humeral Head of Pronator Teres. J. Hand Surg..

[22] Lee H.J., Kim I., Hong J.T., Kim M.S. (2014). Early Surgical Treatment of Pronator Teres Syndrome. J. Korean Neurosurg. Soc..

[23] Danielsson L.G. (1980). Iatrogenic Pronator Syndrome. Scand. J. Plast. Reconstr. Surg..

[24] Morris H.H., Peters B.H. (1976). Pronator syndrome: Clinical and electrophysiological features in seven cases. J. Neurol. Neurosurg. Psychiatry.

[25] Megele R. (1988). Anterior interosseous nerve syndrome with atypical nerve course in relation to the pronator teres. Acta Neurochir..

[26] Mujadzic M., Papanicolaou G., Young H., Tsai T.-M. (2007). Simultaneous Surgical Release of Ipsilateral Pronator Teres and Carpal Tunnel Syndromes. Plast. Reconstr. Surg..

